# Proximal junctional kyphosis in Lenke 5C adolescent idiopathic scoliosis after selective posterior thoracolumbar/lumbar fusion: risk factors and predictive index

**DOI:** 10.1186/s13018-023-04470-5

**Published:** 2024-01-03

**Authors:** Jinyi Bai, Shu Liu, Chen Liu, Yingchuan Zhao, Ming Li

**Affiliations:** 1 Department of Orthopedics,Changhai Hospital of the Second Military Medical University, Shanghai, China; 2 Department of Orthopedics, Shanghai Tenth People’s Hospital, Shanghai, China

## Abstract

**Background:**

Although several studies have reported that selective posterior thoracolumbar/lumbar (TL/L) fusion can yield satisfactory results in Lenke 5C adolescent idiopathic scoliosis (AIS), the proximal junctional kyphosis (PJK) is still a common complication that occurs after surgery. The purpose of this study is to analyse the risk factors for postoperative proximal junctional kyphosis in Lenke 5C patients who underwent selective posterior TL/L fusion and explore whether PJK can be predicted.

**Methods:**

A total of 83 AIS patients with Lenke 5C curves who met the inclusion criteria were analysed. All patients were divided into two groups based on the occurrence of postoperative PJK. Univariate and multivariate analyses were used to identify independent factors significantly associated with PJK, and an PJK index was proposed and verified.

**Results:**

PJK was observed in 27 of 83 (32.5%) patients in the study. Preoperative thoracic kyphosis (TK) and the immediate postoperative proximal junctional angle (PJA) were the primary factors identified by the binary logistic regression analysis. The PJK index was defined as 1.1× preoperative TK + 2.3× immediate postoperative PJA. The receiver operating characteristics curve indicated that the occurrence rate of PJK was 85% and non-occurrence rate was 82% when the PJK index was greater than 42.

**Conclusion:**

Large preoperative TK and a large immediate postoperative PJA play important roles in the development of PJK in Lenke 5C patients treated with selective posterior thoracolumbar/lumbar fusion. The PJK index can be used to predict the occurrence of PJK with high accuracy. To prevent the occurrence of PJK, we should pay attention to the TLK, and preserving more posterior proximal intervertebral elements at the upper instrumented vertebral level would be an important part of corrective surgery; however, moderate correction of the lumbar curve is recommended.

## Introduction

The main purpose of correction surgery for adolescent idiopathic scoliosis (AIS) is to obtain a balanced spine. As the importance of sagittal balance is increasing recognized, sagittal-related complications are attracting more attention [1, 2]. Lenke 5C is a type of AIS mainly characterized by thoracolumbar/lumbar curves (TL/LCs) and minor thoracic curves. Normally, the use of a rigid pedicle screw system enables spine surgeons to perform selective posterior thoracolumbar/lumbar correction for Lenke 5C patients, which can optimize the motion segments of the spine [3, 4].

Adjacent segment disease occurs caudal or cephalad to the rigid instrumented spinal fusion and comprises a variety of pathologies. Proximal junctional kyphosis (PJK) is a common type of adjacent segment disease that occurs after spinal deformity corrective surgery, with an incidence ranging from 7 to 46% [5–7]. Several studies have identified variable risk factors for PJK, including thoracoplasty, fusion level selections, the male sex, and severe preoperative thoracic kyphosis [8–12].

To the best of our knowledge, no previous study has identified the risk factors for PJK with selective posterior thoracolumbar/lumbar fusion in Lenke 5C AIS patients, which may be because of the following reasons: (1) The incidence of PJK in surgically treated Lenke 5C AIS patients is not high enough to attract attention; and (2) PJK is not necessarily associated with poor clinical outcomes and revision surgery in a young age.

Postoperative PJK in adults with spinal deformities is a research hotspot and is associated with several negative effects, including destruction of the vertebrae and ligaments, proximal junctional failure, and need for revision surgery [13, 14]. These complications significantly reduce patient quality of life and place a burden on the patients’ families. For adolescent patients with PJK, growth with age and spinal degeneration may accelerate the progression of PJK. Therefore, having surgeons recognize the risk factors for PJK is important for preventing this complication. The primary purpose of this study was to analyse the risk factors for postoperative PJK in Lenke 5C patients who underwent selective posterior thoracolumbar/lumbar corrective surgery. In addition, an PJK index is proposed and verified to ascertain whether PJK can be predicted.

## Methods and materials

### Patient population

A retrospective study was conducted from a single-centre database. The patients enrolled underwent surgical treatment between April 2016 and March 2017. This study was approved by the Institutional Review Board of our hospital. All patients included in this study provided written informed consent. The inclusion criteria were as follows: (1) AIS patients with Lenke 5C curves who were treated with selective posterior TL/LC corrective surgery by pedicle screws; (2) age younger than 18 years; (3) patients with TL/LCs > 40°; (4) patients with sufficient radiographic parameters and data from standing posteroanterior full-length X-ray films; and (5) patients with at least a 3-year postoperative radiographic follow-up. A total of 83 patients met the inclusion criteria and were included in this study. This study was approved by the Institutional Review Board in our hospital, and all patients in our study written informed consent for the study.

### Data collection

Upright standing anteroposterior and lateral digital radiographs were evaluated at three time points (preoperative, immediate postoperative, and at least 3 years postoperative). On the anteroposterior X-ray film, the minor thoracic curve (MTC) and TL/LC were measured for further analysis. In each lateral radiograph, sagittal parameters were measured, including thoracic kyphosis (TK, the Cobb angle between the upper endplate of T5 and the lower endplate of T12), thoracolumbar kyphosis (TLK, the Cobb angle between the upper endplate of T10 and the lower endplate of L2), lumbar lordosis (LL, the Cobb angle between the upper endplate of L1 and the lower endplate of S1), pelvic tilt (PT, the angle between the vertical line and the line through the midpoint of the sacral plate to femoral axis), sacrum slope (SS, the angle between the horizontal and sacral plates), pelvic incidence (PI, angle subtended by a perpendicular line from the upper endplate of S1 and a line connecting the centre of the femoral head to the centre of the upper endplate of S1), proximal junctional angle (PJA, the angle between the lower endplate of the upper instrumented vertebra (UIV) and the upper endplate of the second supra-adjacent vertebrae), and sagittal vertical axis (SVA, the horizontal offset from the posterosuperior corner of S1 to the vertebral body of C7). The surgical parameters, including the level of the UIV and lower instrumented vertebrae (LIV), were measured and analysed. For this study, all radiographs were obtained twice by each of two independent surgeons, and the average values of each parameter were calculated for the final analyses. Data collection and analysis were performed retrospectively by two senior spine surgeons who were not involved in surgically treating the study patients.

All patients were divided into two groups based on the occurrence of PJK. According to the definition of PJK, two criteria must be considered: (1) a proximal junction sagittal Cobb angle ≥ + 10° and (2) a proximal junction sagittal Cobb angle larger than the preoperative measurement by 10° or more [9, 15].

### Statistical analysis

All of the variables are presented as the means ± standard deviations. Independent two-sample *t*-test analyses and *χ*^2^ tests were used to detect the differences between patients with and without PJK. Factors related to PJK were further investigated by stepwise logistic regression analysis with forward elimination (conditional). Furthermore, the PJK index was developed according to the results of the logistic regression to create a novel predictor for PJK. The sensitivity and specificity of the predictive power of the development of PJK using PJK index were calculated, and the receiver operating characteristic (ROC) curve was drawn.

All data analyses were performed with SPSS 18.0 software (SPSS Inc., Chicago, IL). A *P* value less than 0.05 was considered statistically significant.

## Results

A total of 83 Lenke 5C AIS patients (62 female patients and 21 male patients) who underwent selective posterior thoracolumbar/lumbar corrective surgery were identified and were followed up for a minimum of 3 years. The average age was 13.73 years (ranging from 12 to 18), and the average Risser grade at surgery was 3.69 (ranging from 2 to 5). The mean preoperative Cobb angles of the major TL/LC and minor thoracic curve were 45.46° and 17.36°, respectively. These values were corrected to 11.84 and 10.07 at the last follow-up, which corresponded to correction rates of 74.0% and 42.0%, respectively (Tables 1 and 2).

**Table 1:** General characteristics and radiographic parameters in patients with Lenke 5C AIS (*n* = 83)

Variables	Mean	Standard deviation	Minim	Maxim
Age (years)	13.7	1.4	12	18
Risser	3.7	0.8	2	5
Preoperative TL/LC (°)	45.5	6.1	40	67
Immediate postoperative TL/LC (°)	11.4	4.5	2	23
TL/LC at last follow-up (°)	11.9	4.0	2	22
Preoperative TC (°)	17.4	7.1	5	33
Immediate postoperative TC (°)	9.1	3.5	1	18
TC at last follow-up (°)	10.1	3.6	3	17
Preoperative TK (°)	21.6	8.9	5	38
Postoperative TK (°)	23.0	8.2	10	49
TK at last follow-up (°)	27.0	8.8	14	53
Preoperative TLK (°)	5.5	3.4	− 4	12
Postoperative TLK (°)	7.1	2.8	2	13
TLK at last follow-up (°)	8.6	2.6	3	14
Preoperative LL (°)	50.5	10.6	− 79	− 35
Postoperative LL (°)	54.8	8.0	− 81	− 38
LL at last follow-up (°)	55.4	8.2	− 81	− 38
Preoperative SVA (mm)	− 14.3	28.1	− 67	76
Postoperative SVA (mm)	− 9.3	22.5	− 54	51
SVA at last follow-up (mm)	− 9.6	24.8	− 65	59
Preoperative PJA (°)	4.6	2.1	1	9
Postoperative PJA (°)	6.1	2.4	1	12
PJA at last follow-up (°)	9.2	5.4	2	23
Preoperative PT (°)	10.9	6.5	1	31
Postoperative PT (°)	8.4	5.7	1	26
PT at last follow-up (°)	11.6	6.7	2	34
Preoperative SS (°)	35.5	6.4	23	48
Postoperative SS (°)	38.1	5.7	21	51
SS at last follow-up (°)	35.1	6.7	23	48
Preoperative PI (°)	46.4	6.0	36	59
Postoperative PI (°)	46.5	5.5	35	58
PI at last follow-up (°)	46.2	5.5	37	58
Preoperative PI-LL	− 4.2	10.2	− 27	15
Postoperative PI-LL	− 8.3	8.2	− 31	9
PI-LL at last follow-up	− 9.2	8.5	− 29	10

*TL/LC* major thoracolumbar/lumbar curve, *TC* minor thoracic curve, *TK* thoracic kyphosis, *TLK* thoracolumbar kyphosis, *LL* lumbar lordosis, *PT* pelvic tilt, *SS* sacral slope, *PI* pelvic incidence, *PJA* proximal junctional angle, and *SVA* sagittal vertebrae axis

**Table 2:** Pre- and postoperative coronal and sagittal radiographical parameters in patients with Lenke 5C AIS (*n* = 83)

	Preoperative	Immediate postoperative	Last follow-up	*P* (pre vs. Im-Post)	*P* (pre vs. LFU)	*P* (Im-Post vs. LFU)
Coronal plane						
TC (°)	17.4 ± 7.1	9.1 ± 3.5	10.1 ± 3.6	< 0.001	< 0.001	0.002
TL/LC (°)	45.5 ± 6.1	11.4 ± 4.5	11.8 ± 4.0	< 0.001	< 0.001	0.035
Sagittal plane						
TK (°)	21.6 ± 8.9	23.0 ± 8.2	27.0 ± 8.8	0.080	< 0.001	< 0.001
TLK (°)	5.5 + 3.4	7.1 ± 2.8	8.6 ± 2.6	< 0.001	< 0.001	< 0.001
LL (°)	50.5 ± 10.6	54.8 ± 8.0	55.4 ± 8.2	< 0.001	< 0.001	0.052
PJA (°)	4.6 ± 2.1	6.1 ± 2.4	9.2 ± 5.4	< 0.001	< 0.001	< 0.001
SVA (mm)	− 14.0 ± 28.1	− 9.3 ± 22.5	− 9.6 ± 24.8	0.040	0.630	0.658
Pelvic parameters						
PT (°)	10.9 ± 6.5	8.4 ± 5.7	11.6 ± 6.7	< 0.001	0.188	< 0.001
SS (°)	35.5 ± 6.4	38.1 ± 5.7	35.1 ± 6.7	< 0.001	0.583	< 0.001
PI (°)	46.4 ± 6.0	46.5 ± 5.5	46.2 ± 5.5	0.685	0.587	0.431

*TC* thoracic curve, *TL/LC* thoracolumbar/lumbar curve, *TK* thoracic kyphosis, *TLK* thoracolumbar kyphosis, *LL* lumbar lordosis, *PT* pelvic tilt, *SS* sacral slope, *PI* pelvic incidence, *PJA* proximal junctional angle, and *SVA* sagittal vertebrae axis

At the last follow-up, 27 patients developed PJK; the incidence of PJK was 32.5%. No patients in the cohort underwent revision surgery due to the occurrence of PJK.

### Univariate analysis of the clinical and radiographic factors

The results are illustrated in Tables 3 and 4. All patients were divided into two groups based on the occurrence of postoperative PJK: a PJK group and a non-PJK group. No significant differences existed between the groups with respect to sex and Risser grades. The patients with PJK were older than the patients without PJK.

**Table 3:** Comparison analysis of the demographic and surgical-related data between the two groups

Variable	PJK	Non-PJK	*P*
Age (years)	14.22 ± 1.45	13.50 ± 1.36	0.029
Sex			0.324
Female	22	40	
Male	5	16	
Risser	3.48 ± 0.80	3.79 ± 0.78	0.103
UIV			0.266
T9	7	24	
T10	12	14	
T11	7	14	
T12	1	4	
LIV			0.290
L3	5	4	
L4	20	48	
L5	2	4

**Table 4:** Comparison analysis of the radiological parameters between the two groups

	PJK	Non-PJK	*P*
Preoperative radiographical factors			
TC (°)	15.74 ± 5.45	18.14 ± 7.68	0.106
TL/LC (°)	44.04 ± 2.43	46.14 ± 7.18	0.052
TK (°)	27.89 ± 7.21	18.57 ± 8.06	< 0.001
TLK (°)	5.93 ± 2.81	5.25 ± 3.59	0.394
LL (°)	53.15 ± 10.52	49.29 ± 10.45	0.119
PT (°)	10.70 ± 6.29	10.95 ± 6.67	0.875
SS (°)	35.22 ± 5.63	35.64 ± 6.76	0.780
PI (°)	45.93 ± 6.28	46.55 ± 5.83	0.655
PJA (°)	5.67 ± 2.17	4.52 ± 2.10	0.024
SVA (mm)	− 3.10 ± 32.50	− 19.31 ± 24.23	0.027
PI-LL	− 7.22 ± 11.82	− 2.73 ± 9.13	0.061
Immediate postoperative radiographical factors			
TC (°)	9.48 ± 4.71	8.96 ± 2.72	0.600
TL/LC (°)	11.30 ± 4.50	11.39 ± 4.50	0.927
TK (°)	25.26 ± 7.37	21.86 ± 8.39	0.076
TLK (°)	7.70 ± 2.37	6.79 ± 2.91	0.158
LL (°)	56.00 ± 9.46	54.14 ± 7.22	0.325
PT (°)	8.00 ± 5.704	8.61 ± 5.75	0.653
SS (°)	38.33 ± 7.06	38.00 ± 5.05	0.827
PI (°)	46.11 ± 6.10	46.61 ± 5.21	0.702
PJA (°)	8.11 ± 1.91	5.11 ± 2.02	< 0.001
SVA (mm)	− 3.18 ± 20.20	− 12.21 ± 23.06	0.086
PI-LL	− 9.89 ± 10.06	− 7.54 ± 7.01	0.220
Radiographical factors at the last follow-up			
TC (°)	9.04 ± 3.37	10.57 ± 3.69	0.072
TL/LC (°)	11.37 ± 4.19	12.07 ± 3.87	0.454
TK (°)	29.15 ± 8.19	26.00 ± 8.97	0.128
TLK (°)	8.89 ± 2.52	8.52 ± 2.67	0.551
LL (°)	56.19 ± 10.07	54.96 ± 7.20	0.528
PT (°)	10.63 ± 4.98	12.00 ± 7.34	0.384
SS (°)	35.72 ± 6.36	34.84 ± 6.96	0.578
PI (°)	46.36 ± 4.94	46.05 ± 5.77	0.814
PJA (°)	16.26 ± 2.65	5.82 ± 1.85	< 0.001
SVA (mm)	− 0.18 ± 22.08	− 14.17 ± 24.91	0.015
PI-LL	− 9.83 ± 9.88	− 8.91 ± 7.77	0.647
HRQOL at the last follow-up			
Pain	4.22 ± 0.64	4.29 ± 0.49	0.621
Appearance	3.63 ± 0.74	4.45 ± 0.69	< 0.001
Activity	4.26 ± 0.45	4.34 ± 0.61	0.502
Mental health	4,22 ± 0.42	4.32 ± 0.47	0.340
Satisfaction	4.04 ± 0.52	4.18 ± 0.39	0.167
Total score	4.07 ± 0.40	4.31 ± 0.31	0.004

*TC* thoracic curve, *TL/LC* thoracolumbar/lumbar curve, *TK* thoracic kyphosis, *TLK* thoracolumbar kyphosis, *LL* lumbar lordosis, *PT* pelvic tilt, *SS* sacral slope, *PI* pelvic incidence, *PJA* proximal junctional angle, and *SVA* sagittal vertebrae axis

Compared with patients without PJK, the patients with PJK had a significantly larger TK (*P* < 0.001) before surgery. The PJK group had a significantly larger PJA before surgery, immediately after surgery, and at the last follow-up (*P* = 0.024, *P* < 0.001, and *P* < 0.001). The SVAs were significantly different between the PJK group and the non-PJK group both before surgery and at the last follow-up (*P* = 0.027 and *P* = 0.015). No significant differences in the distributions of UIV and LIV were found between the two groups.

At the final follow-up, patients with PJK had worse scores of appearances (3.63 ± 0.74 vs. 4.45 ± 0.69, *P* < 0.001) than patients without non-PJK; however, there were no significant differences in the scores of the other SRS-22 domains between the two groups (all *P* > 0.05).

**Fig. 1:**
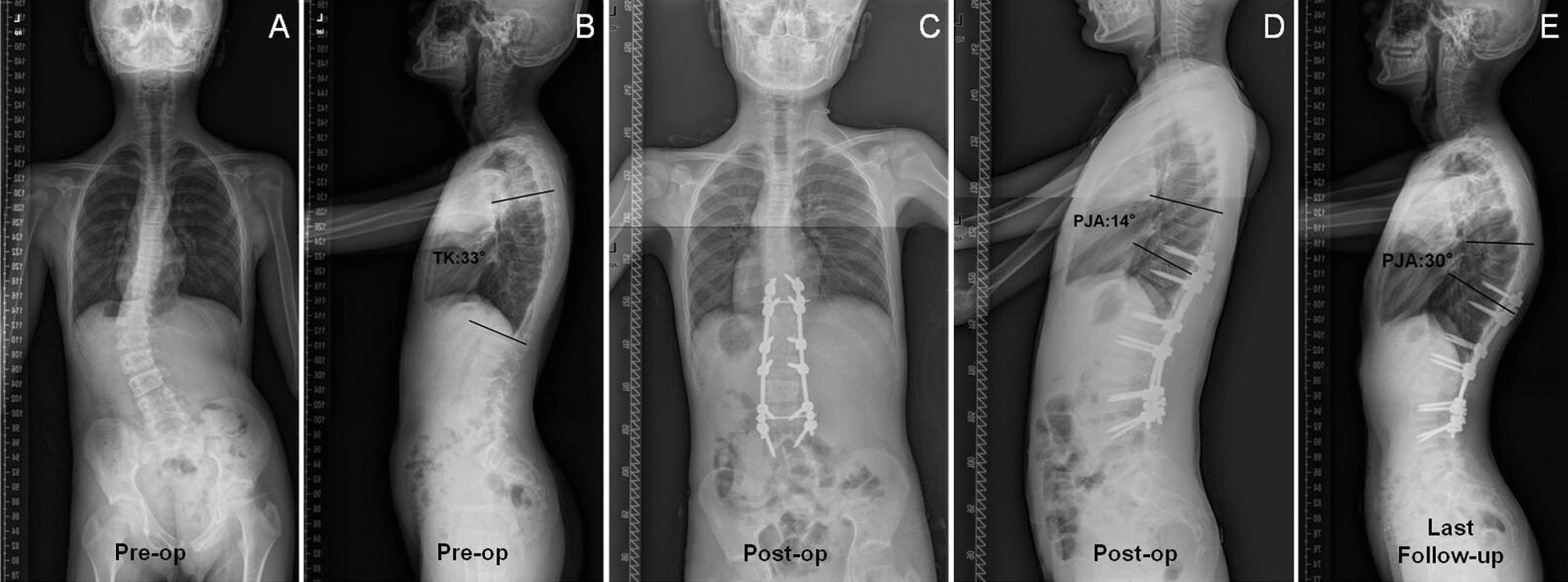
Proximal junctional kyphosis (PJK) group: A 16-year-old male patient with Lenke 5CN adolescent idiopathic scoliosis (AIS). The preop TK was 33°. The PJA was 14° and 30° immediate postoperation and at follow-up, respectively. The PJK index was 68.5 > 42

**Fig. 2:**
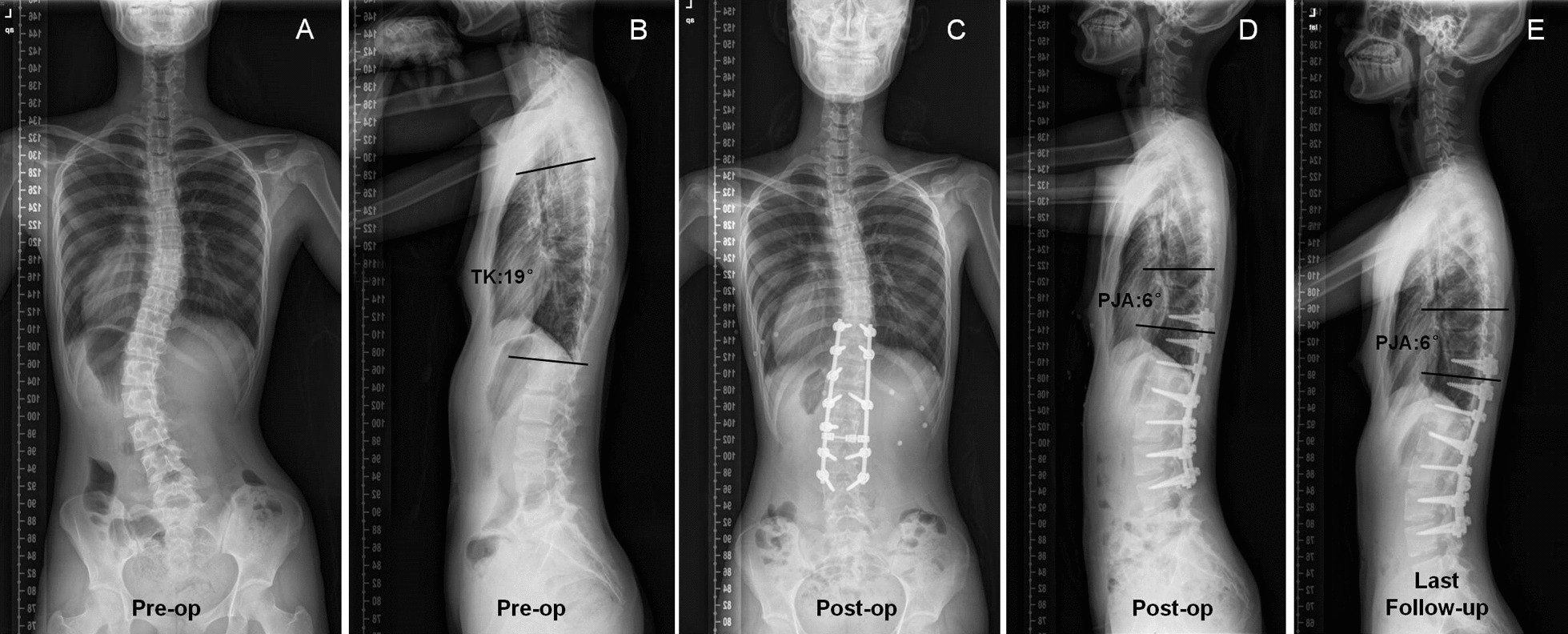
Non-PJK group: A 13-year-old female patient with Lenke 5CN AIS. The preop TK was 19°. The PJA was 6° and 6° immediate postoperation and at follow-up, respectively. The PJK index was 34.7 < 42

Two typical cases are shown in Figs. 1 and 2.

### Binary logistic analysis of the related factors

The significant preoperative and immediate postoperative factors identified by independent two-sample *t*-tests and ANOVA were further tested by a stepwise logistic regression analysis. The preoperative TK and immediate postoperative PJA were the primary factors included in the equation (OR 1.119, CI 1.030–1.217, *P* = 0.008 and OR 2.279, CI 1.461–3.557, *P* < 0.001, respectively), as summarized in Table 5. According to the regression equation, the PJK index is defined as 1.1* preoperative TK + 2.3* immediate postoperative PJA.

**Table 5:** Binary logistic regression analysis of the risk factors for PJK

Variables	*B*	S.E	Wald	*df*	*P*	Exp (B)	95% CI
Preoperative TK	0.133	0.043	6.992	1	0.008	1.119	1.030–1.217
Immediate postoperative PJA	0.824	0.227	13.170	1	< 0.001	2.279	1.461–3.557

*95% CI* 95% confidence interval, *OR* odds ratio, and *S.E.* standard error

### ROC curves

**Fig. 3:**
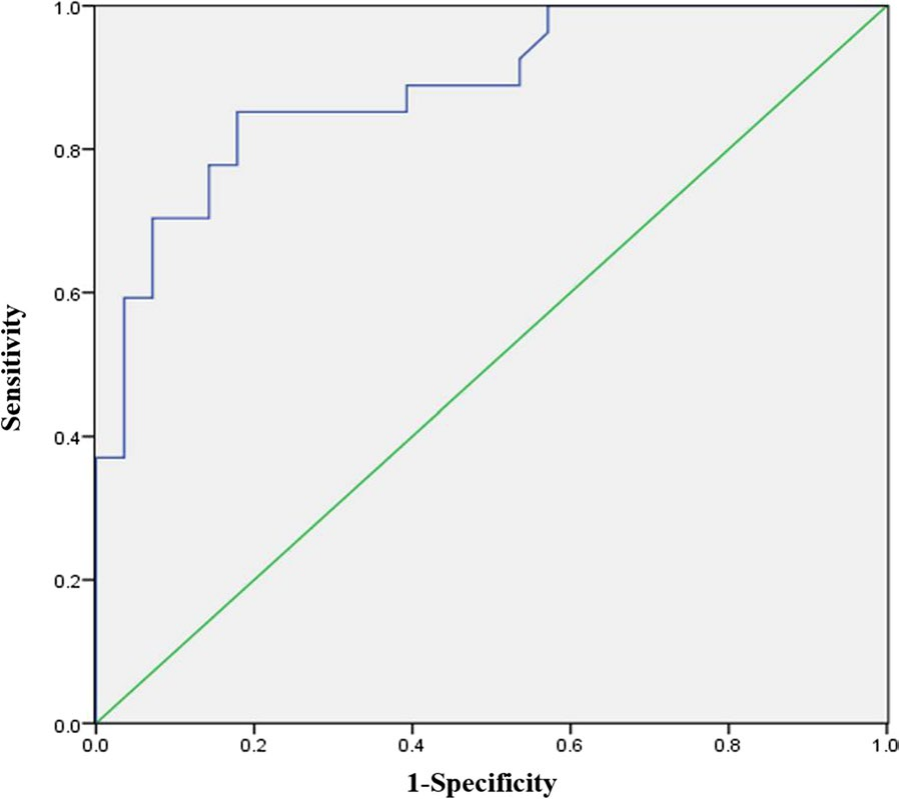
ROC curves of predictive power of the occurrence of PJK using PJK index

Based on the ROC curves, the optimal cutoff values for the PJK index were calculated to be 42. If the PJK index was greater than 42, the occurrence rate of PJK was 85%, and the non-occurrence rate was 82% (Fig. 3).

## Discussion

Selective thoracolumbar/lumbar fusion (STLF/SLF), including both the anterior and posterior approaches, is increasingly recognized as the ideal surgical treatment for Lenke 5C AIS patients [16, 17]. The previous studies reported that both anterior and posterior selective fusions of the main curve of Lenke 5C patients could obtain good spontaneous correction of the thoracic curve [18]. With the application of three-dimensional spinal instruments, the development of PJK after selective posterior fusion has attracted increasing interest. To the best of our knowledge, only a few relevant reports exist on postoperative PJK in Lenke 5C AIS patients.

PJK is a specific imaging change at the transition between the fused and mobile spinal segments, usually due to the stress changes in proximal fixation. In recent years, PJK has been found to not only be an imaging change but also to produce clinical symptoms that affect patient quality of life and sometimes even requires surgical revision, especially in adults [13, 14]. For surgically treated adolescents with PJK, growth with age and spinal degeneration may accelerate PJK progression. However, it remains unclear whether PJK can be predicted. Therefore, we determined the risk factors for PJK in Lenke 5C patients who underwent selective posterior thoracolumbar/lumbar fusion by pedicle screws and developed the PJK index to predict the rate of occurrence of postoperative sagittal imbalance.

The univariate analysis results indicated that the patients in the PJK group had a larger preoperative TK and preoperative PJA and SVA than patients with non-PJK, which was similar to the results of other studies [6, 19]. There are several reasons could explain why the Lenke 5C AIS patients with a larger preoperative TK and preoperative PJA and SVA were more likely to suffer from PJK. First, posterior selective fusion can achieve spontaneous correction of unfused thoracic curves in Lenke 5C AIS. A larger preoperative TK and preoperative PJA and SVA accompanied by spontaneous correction of unfused thoracic curves could generate proximal buckling stress, resulting in PJK. Second, surgeons tend to disrupted posterior ligament at UIV/UIV + 1. For Lenke 5C patients treated by selective posterior thoracolumbar/lumbar fusion, UIV was located at thoracolumbar region which is junction of TK and LL. As a result, the range of motion at this level might increase, leading to postoperative sagittal imbalance [20].

In terms of postoperative parameters, the immediate postoperative PJA was significantly greater in the PJK group than the non-PJK group. It is comprehensible that a greater immediate postoperative PJA will cause postoperative sagittal imbalance, and inadequate restoration of the proximal junctional region might lead to PJK. In addition, PJA and SVA at the last follow-up were significantly greater in PJK group than the non-PJK group, which were consistent with the definition of PJK [9, 15].

The multivariate analysis results showed that the preoperative TK was significantly associated with postoperative PJK, which has been observed in the previous studies [9, 10, 21, 22]. Maruo et al. [21] retrospectively reviewed 90 adult spinal deformity (ASD) patients and revealed that a preoperative TK larger than 30° was a predictor of PJK. However, only ASD patients who underwent long instrumented fusion to the sacrum were included in the study. Kim et al. [10] indicated that AIS patients with a large preoperative sagittal thoracic Cobb angle (T5–12) were more likely to experience PJK during an average follow-up of 7.3 years. The authors postulated that a substantial TK correction may lead to PJK. In another study by Kim [9], a large preoperative TK angle and a large reduction in the immediate postoperative TK angle were found to be significantly correlated with PJK. Therefore, Kim et al. attributed the occurrence of PJK to the substantial change in TK. Mendoza-Lattes et al. [22] found significant differences between TK and LL in PJK patients (*P* = 0.012) and concluded that PJK developed in patients with a TK that remained greater in magnitude relative to LL. In our study, we retrospectively analysed Lenke 5C AIS patients with selective posterior thoracolumbar/lumbar fusion. The multivariate analysis indicated that the preoperative TK was a main risk factor for PJK. This result expands upon those from the previous studies and provides additional evidence that a large preoperative TK may play an important role in causing PJK, even in Lenke 5C type patients with selective posterior thoracolumbar/lumbar fusion. Our results showed that the LL increased significantly after surgery. The larger LL indicated that patients had a trend of backward incline. Therefore, to maintain a global sagittal balance, a compensatory mechanism that increases TK to adapt to the increased LL may exist. For patients with large preoperative TK angles, this increase in TK may further aggravate the concentration of stress in the proximal junction, leading to the occurrence of PJK [11]. However, PJK that might cause forward incline of body occurred to compensate for sagittal balance. Therefore, we recommend that moderate correction of the lumbar curve should be adjusted to TK.

Moreover, our study also revealed that the immediate postoperative PJA was an independent risk factor for PJK. Lee et al. [6] evaluated 106 patients with idiopathic scoliosis and reported that a preoperative PJA larger than 5° was a risk factor for PJK. Yan’s study [23] found that for patients with degenerative scoliosis, PJK patients had a much larger postoperative PJA than those without PJK (13.42 ± 6.65 vs. 7.27 ± 4.28, *P* = 0.007). Similarly, Maruo et al. [21] identified a preoperative PJA larger than 10° as a risk factor for PJK (58% vs*.* 32%, *P* = 0.016). During selective posterior fusion of the thoracolumbar/lumbar curve, most of thoracic segments were left unfused whereas most of lumbar segment were fused. UIV was located at thoracolumbar region in our study, and we did not find that UIV was correlated with the prevalence of PJK. Thoracolumbar region is also the area that transfers force from thoracic spine to lumbar spine. Thus, the instrumentation terminated proximally in the lower thoracic spine, and patients frequently compensate for a more kyphotic posture through the remaining mobile thoracic spine. However, the reciprocal change in the non-fused thoracic segments is limited. Thus, we speculated that the trunk can adjust to achieve a new overall balance through changes in the thoracolumbar region and PJA would change. In our study, patients with PJK had significantly larger preoperative TK and SVA, while the preoperative PJA showed no difference between the two groups. Furthermore, patients with PJK had significantly larger PJA immediately after surgery and at the last follow-up, while immediate postoperative TK and SVA showed no difference between the two groups immediately after surgery and at the last follow-up, verifying our speculation that PJK was a compensatory mechanism for sagittal alignment, and the thoracolumbar region and PJA would change. Furthermore, selective posterior fusion will damage the posterior soft tissue and ligament structure, which generates an increased PJA and proximal buckling stress [24, 25]. Consequently, an increased postoperative PJA can be a marker of sagittal malalignment and results in PJK in the follow-up. Therefore, the thoracolumbar kyphosis plays a key role in PJK, and moderate correction of TLK should be performed to match the TK and LL, thereby reducing the risk of PJK. A study by Anderson et al. in 2009 indicated that supraspinous and interspinous ligament transection resulted in a marginally significant loss of motion segment flexion stiffness [26]. Cammarata et al. [24] studied biomechanical spine models of six patients with adult scoliosis and found that the bilateral complete facetectomy, the posterior ligaments resection, and the combination of both increased the proximal junctional kyphotic angle (respectively, by 10%, 28%, and 53%). In the present study, our results indicate that the patients with lager immediate PJA are more likely to develop PJK. In clinical practice, it is, therefore, imperative that we aim for preserving more posterior proximal intervertebral elements at the upper instrumented vertebral level to reduce the risk of PJK. Further studies will be required to investigate how to control the PJA and decrease proximal buckling stress.

None of the demographic and surgical-related parameters were found to be associated with the occurrence of PJK except age. However, the multivariate analysis did not identify age as an independent risk factor. We believe that this result was an accidental phenomenon. Because the age of AIS patients is relatively concentrated to adolescence, there is no age-associated degeneration or other factors that lead to PJK.

Based on the binary logistic regression analysis, we defined PJK index as 1.1 *preoperative thoracic kyphosis + 2.3 * immediate postoperative proximal junctional angle. The results of the ROC showed that the optimal cutoff values for the PJK index were calculated to be 42. If the PJK index was greater than 42, the occurrence rate of PJK was 85%, and the non-occurrence rate was 82%. These results indicated that PJK is predictable, and the PJK index was suitable for this purpose.

However, there are some limitations in this study. First, our study is a single-centre study. Second, a longer follow-up is needed to further explore the mechanism of PJK. Therefore, larger multicentre studies with longer follow-up periods should be conducted on PJK in AIS patients.

## Conclusion

Large preoperative TK and a large immediate postoperative PJA play important roles in the development of PJK in Lenke 5C patients treated with selective posterior thoracolumbar/lumbar fusion. The PJK index can be used to predict the occurrence of PJK with high accuracy. To prevent the occurrence of PJK, we should pay attention to the TLK, and preserving more posterior proximal intervertebral elements at the upper instrumented vertebral level would be an important part of corrective surgery; however, moderate correction of the lumbar curve is recommended.

## Data Availability

None.
